# Supplementary material for: Splice-Junction-Based Mapping of Alternative Isoforms in the Human Proteome
Source: Cell Rep. Author manuscript; Available in PMC 2020 Jan 15. (PMC6961840; doi:10.1016/j.celrep.2019.11.026)
Supplement: 3 [file NIHMS1546469-supplement-3.zip › DF2/PXD006675/PulmonaryArtery-263-P56159-VVPFISVEHIPK.pdf]

A

Predicted sequence disorder and sequence features of P56159

Peptide: VVPFISVEHIPK Junction: sp|P56159|GFRA1_HUMAN|ENSG00000151892|SE2|34693|chr10|116125557|116211645|−2|r8|T1 TrNovel: FALSE

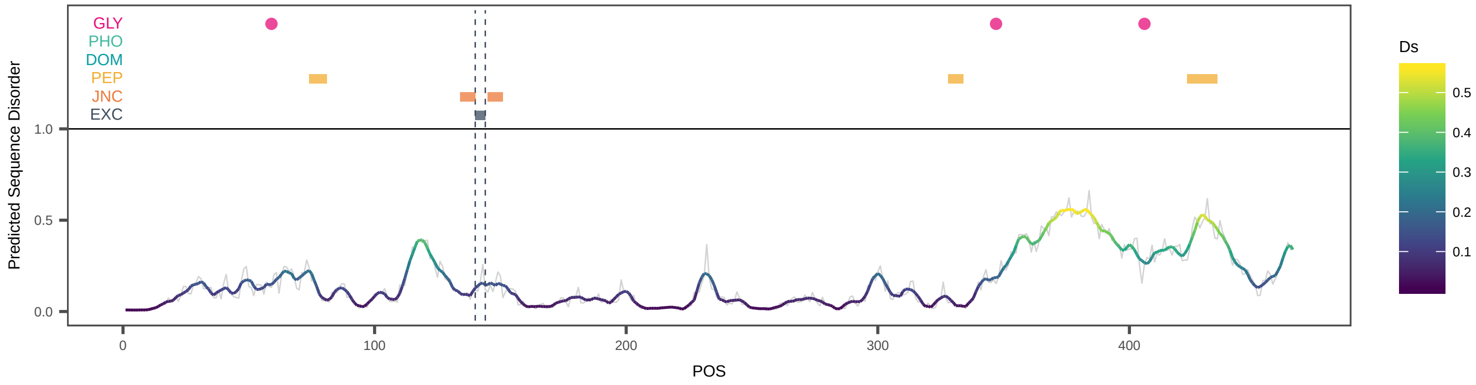

B

Distribution of sequence disorder in excised
vs. mapped and non−excised regions of protein

M−W P−value vs. mapped: 0.481 vs. non−excised: 0.483

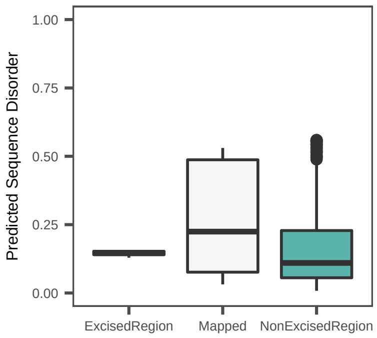

C
